# Exosomes: their role and therapeutic potential in overcoming drug resistance of gastrointestinal cancers

**DOI:** 10.3389/fonc.2025.1540643

**Published:** 2025-05-13

**Authors:** Jiulian Liu, Shanyu Gao, Xiaoming Liu, Jiaxin Dong, Dingwei Zhen, Tong Liu

**Affiliations:** ^1^ Department of Anorectal Surgery, The Fourth People’s Hospital of Jinan, Jinan, China; ^2^ Department of Anorectal Surgery, Affiliated Hospital of Shandong University of Traditional Chinese Medicine, Jinan, China; ^3^ Department of Health Care, Affiliated Hospital of Shandong University of Traditional Chinese Medicine, Jinan, China; ^4^ College of Traditional Chinese Medicine, Shandong University of Traditional Chinese Medicine, Jinan, China; ^5^ First Clinical Medical College, Shandong University of Traditional Chinese Medicine, Jinan, China; ^6^ Department of Clinical Laboratory, Qilu Hospital of Shandong University, Jinan, China; ^7^ Department of Clinical Laboratory, Shandong Engineering Research Center of Biomarker and Artificial Intelligence Application, Jinan, China

**Keywords:** exosomes, gastrointestinal cancer, drug resistance, biological function, clinical application

## Abstract

Gastrointestinal cancers are prevalent malignant neoplasms in clinical medicine. The development of drug resistance in gastrointestinal cancers result in tumor recurrence and metastasis and greatly diminish the efficacy of treatment. Exosomes, as the shuttle of intercellular molecular cargoes in tumor micro-environment, secreted from tumor and stromal cells mediate drug resistance by regulating epithelial-mesenchymal transition, drug efflux, stem-like phenotype and cell metabolism. Meanwhile, exosomes have already received tremendous attention in biomedical study as potential drug resistant biomarkers as well as treatment strategy in gastrointestinal cancers. Primary challenge to implement this potential is the ability to obtain high-grade exosomes efficiently; however, exosomes lack standard protocols for their processing and characterization. Furthermore, this field suffers from insufficient standardized reference materials and workflow for purification, detection and analysis of exosomes with defined biological properties. This review summarize the unique biogenesis, composition and novel detection methods of exosomes and informed the underlying correlation between exosomes and drug resistance of gastrointestinal cancers. Moreover, the clinical applications of exosomes are also summarized, might providing novel therapy for the individual treatment of gastrointestinal cancers.

## 1 Introduction

Gastrointestinal (GI) cancers, mainly including esophageal cancer (EC), gastric cancer (GC), hepatocellular carcinoma (HCC), pancreatic cancer (PC), and colorectal cancer (CRC), seriously threaten human life and health. Recent studies demonstrated that GI cancers accounted for 26% (4.8 million) of all cancer cases worldwide in 2018, and there will likely be 7.5 million new cases of GI cancers diagnosed worldwide, with 5.6 million deaths from the disease in 2024 (1). Most GI cancers, once diagnosed, have already progressed to advanced stages because of the insidious onset, the limited effect of non-invasive screening indicators, as well as the pain and high cost of invasive procedures (2). Chemotherapy is the primary conventional treatment for patients with advanced GI tumors or those who are intolerant to surgery (3). Nevertheless, drug resistance has the effect of significantly reducing the efficacy of chemotherapy, which is closely linked to the unfavorable prognosis. Recent researches have proposed that tumor drug resistance is associated with a variety of mechanisms, such as enhanced proliferation and efflux of drugs, gene mutations and epigenetic alterations, increased DNA repair capacity, and elevated metabolism of xenobiotics, etc. (4) However, the exact mechanism of tumor drug resistance is still unclear. Thus, it is imperative to learn more about the molecular mechanisms underlying drug resistance and find valuable biomarkers that can predict and track the response of therapy to improve the outcome of GI cancers.

Previous studies indicated that exosomes in the tumor microenvironment (TME) play an essential role in tumorigenesis, metastasis and drug resistance (5). As nano-sized vesicles carrying information, exosomes could influence the phenotypes of both the parent and target cells during their transit (6). On the one hand, normal cells secrete exosomes to maintain the normal operation of the body. On the other hand, exosomes derived from tumor cells, cancer-associated fibroblasts (CAFs), or mesenchymal stem cells can target recipient cells to establish a fertile microenvironment, suppress the immune system, and remove chemotherapy drugs, thereby promoting cancer progression and drug resistance (7).

In this review, we summarized the most current progress on the correlation between exosomes cargo with GI cancer drug resistance. Meanwhile, we also discussed the potential value of exosomes as cancer biomarkers predicting therapeutic response and as therapeutic strategies in clinical medicine.

## 2 Biogenesis and composition of exosomes

**Figure 1:**
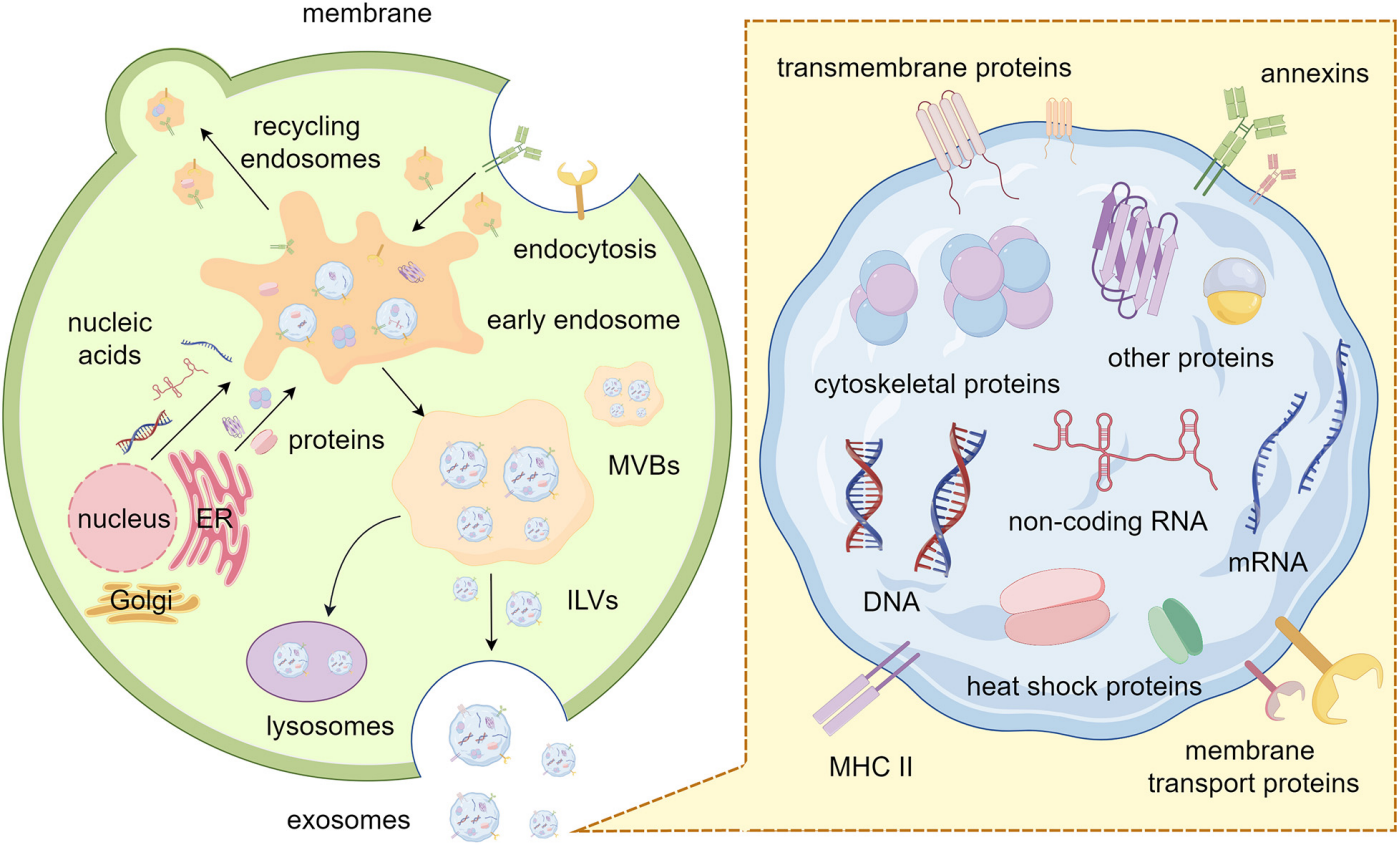
Biogenesis and composition of exosomes. The process of plasma membrane budding inward generates early endosomes. These endosomes are transformed into MVBs after sequestering specific cargos. Finally, the ILVs inside the MVBs are released outside the cell as exosomes. Exosomes contain various nucleic acids, proteins, and lipids, each with specific function. By Figdraw.

Exosomes, typically 30 to 150 nm in diameter, are encapsulated within a single membrane. It has been proved that a variety of nucleic acids, proteins, lipids and glycoconjugates are loaded into exosomes with the regulation of the endosomal sorting complexes required for transport (ESCRT)-dependent or other ESCRT-independent pathways. Exosomes can be secreted by nearly all types of cells and have been found in a lot of body fluids, such as plasma, serum, urine, semen, saliva, bronchial fluid, cerebral spinal fluid, breast milk, tears, etc. (8) The first step in exosome biogenesis is the formation of early exosomes as a result of inward budding of the plasma membrane. More importantly, there are two pathways for early endosome progression: returning to the plasma membrane as “recycling endosomes” or transforming into multivesicular bodies (MVBs). MVBs are formed by inward budding of the limiting membrane of early endosomes and sequestering specific proteins, lipids and cytosolic components. This process results in MVBs containing multiple intraluminal vesicles (ILVs) that package cargo. Eventually, MVBs merge with lysosomes to degrade cargo or with the plasma membrane to release ILVs, known as exosomes, into the extracellular space (9–11). (**Figure 1**).

Exosome contents are heterogeneous, they largely reflect the composition of the donor cells and constantly alter in reaction to external stimuli (12). Firstly, exosomes contain abundant nucleic acids (e.g., DNA, mRNA, and non-coding RNA), some of which have already been identified for delivery to recipient cells, potentially regulating their gene expression and influencing cell progression (13). Notably, mitochondrial DNA could also be sorted into exosomes (14). In mechanism, the interaction between MVBs and mitochondria is facilitated by the activation of PINK1 due to mitochondrial damage. Meanwhile, mGluR3 enhances the invasion of tumor cells by facilitating forward transport of these exosomes coupled with Rab27 protein. Furthermore, RNA-binding proteins (RBPs) with specific RNA-binding domain sequences perform an important role in the sorting of exosomal RNAs (15). Many RBPs (e.g., Ago2, FMR1 and hnRNPA2B1) play crucial roles in the sorting of exosomal RNAs in various cell types (16–20). For instance, the functions of hnRNPA1, SNF8 and hnRNPA2B1 were necessary for the loading process of noncoding RNAs circNEIL3, circRHOBTB3, lncARSR, and LNMAT2 (21, 22). Additionally, the formation of non-membrane carriage like RNA granules (such as stress granules and P-bodies) in cells occurs through a widespread physical process called liquid-liquid phase separation (LLPS) (23). And LLPS-mediated condensate formation relies heavily on the crucial roles played by RBPs and RNAs (23, 24). Emerging evidence indicates that some common proteins can also be found in diverse exosomes, such as transmembrane proteins (e.g., CD9, CD63, and CD81) from the tetraspanin family, programmed cell death 6-interacting proteins (PDC D6IPs), tumor susceptibility gene 101 (Tsg101) proteins, and major histocompatibility complex (MHC) class II molecules. Other exosomal proteins include cytoskeletal proteins (e.g., actin and tubulin), membrane transport proteins, heat shock proteins (e.g., HSP60, HSP70, and HSP90), and annexins (25). Studies also have shown that according to the biogenetic process of exosomes, it is generally accepted that the lipid composition in exosomes should roughly match the composition of the lipid bilayer, sphingomyelin, cholesterol, ganglioside GM3, desaturated lipids, phosphatidylserine, and ceramide are lipid classes commonly found in exosomes (26). These lipids are mainly involved in maintaining the exosomal morphology and intercellular signaling (27). Of note, cargoes packaged within exosomes provide additional traits for their identification and function and supply a potential new tool for liquid biopsy and bio-therapeutics in clinical medicine.

## 3 Separation and detection of exosomes

The emergence of a separation method with high recovery, high purity and high throughput is significant for exosome research at this stage, but it is often costly, inefficient, and contaminated with co-precipitated protein aggregates. Based on the physical and biological characteristics of exosomes, various conventional separation methods have been explored, such as ultra-centrifuge, size-exclusion chromatography, immunoaffinity, polymer precipitation, and some other exosome separation kits (ExoQuick™ and Total Exosome Isolation) (28, 29). Each of them has its own unique advantages and insurmountable disadvantages, even ultra-centrifuge, once considered the “gold standard” for exosome separation (30).

**Figure 2:**
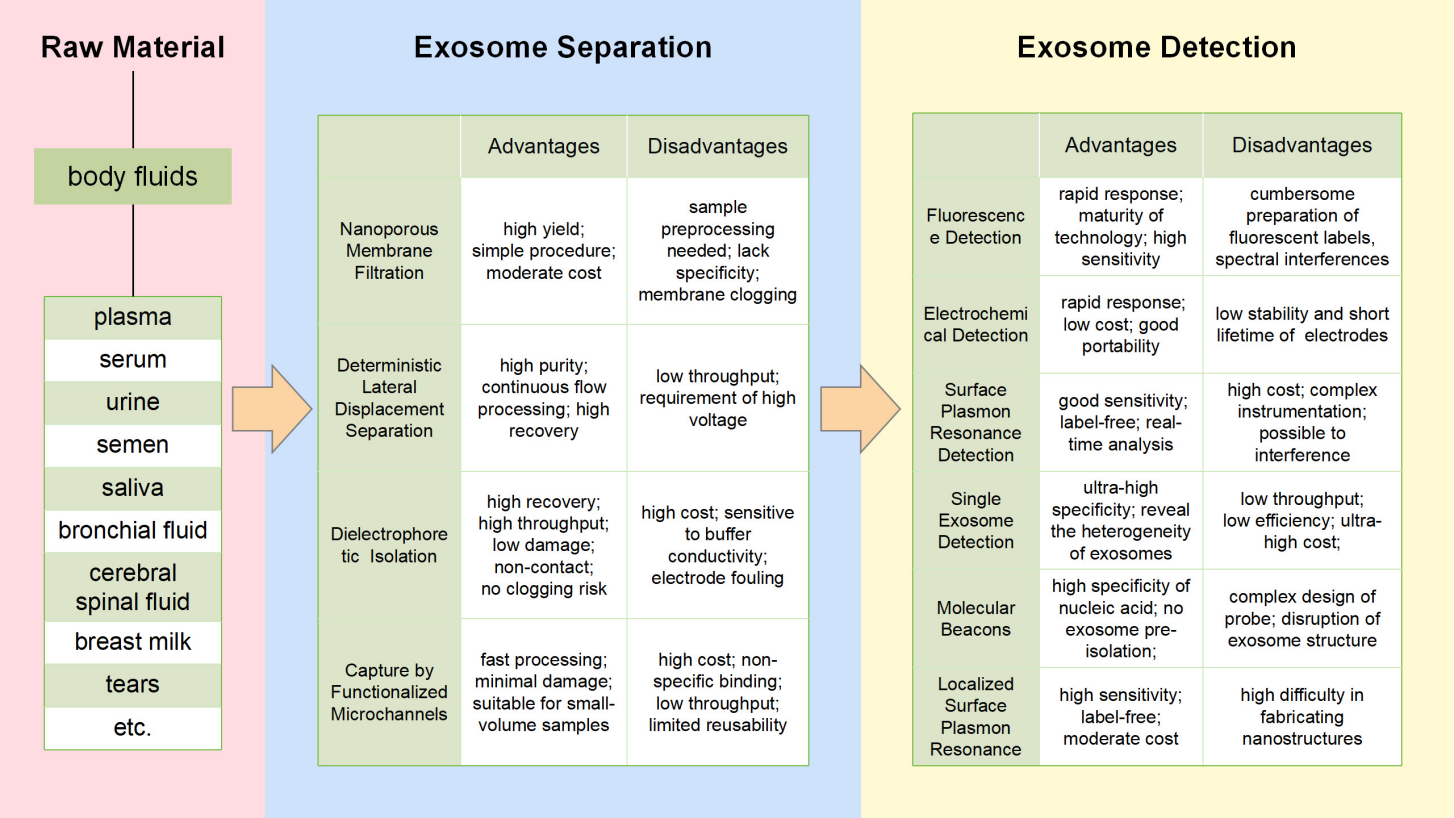
Microfluidics technologies for exosomes separation and detection flowchart. The figure shows the common methods for exosome isolation and detection based on microfluidics, and lists the respective advantages and disadvantages of each method.

In view of the fact that a single method cannot perfectly separate exosomes, many researchers have attempted to combine multiple methods. Therefore, the new separation techniques are emerging, like microfluidics. As a rising star in exosome isolation, microfluidics technology is more like an integrated platform, which functions with different common exosomal separation methods according to the corresponding properties of exosomes. Of note, microfluidics technology can achieve portability, reconfigurability, high throughput, and automation, and promises to be an ideal tool for precision medicine (31) (**Figure 2**).

### 3.1 Exosome separation based on microfluidics

Exosome separation section of **Figure 2** summarizes four common exosome processing techniques: Nanoporous Membrane Filtration, Deterministic Lateral Displacement (DLD) Separation, Dielectrophoretic (DEP) Isolation and Capture by Functionalized Microchannels. These methods vary in the resulting exosome isolation and yield. For each technique, we discuss the physicochemical basis of exosome purification.

#### 3.1.1 Nanoporous membrane filtration

Nanoporous membrane filtration was created by an integrated double-filtration microfluidic system for the separation and enrichment of exosomes from urine, and then quantitatively detected exosomes using a chip Enzyme-Linked Immunosorbent Assay (ELISA). Therefore, urine exosomes can be isolated, enriched, and quantified by the device to aid in the screening process for bladder cancer (32). Liu et al. designed and successfully developed an isolation tool called Exosome Total Isolation Chip (ExoTIC), which is simple, fast, economical, scalable, and high-yield for isolating exosomes from various body fluids of cancer patients (33). ExoTIC provides a lot of exosomes for downstream analysis and creates a standard microenvironment for subsequent research without being separated into separate modules.

#### 3.1.2 Deterministic lateral displacement separation

DLD embeds the gradient column array in a microfluidic device to continuously separate suspended particles by diameter (34). The DLD clutch-column array platform has been applied to isolate circulating tumor cells (CTCs), and a method for sensitive detection of non-fluorescent tags with micron-scale protein and polymer vesicle properties using DLD clutch-column arrays has been established (35, 36). Moreover, the Exodisc (a microfluidic tangential flow filtration device) could effectively separate exosomes from body fluid, resulting in a higher yield of exosomes than traditional technologies (37).

#### 3.1.3 Dielectrophoretic isolation

The DEP device induces polarized particles to move in the non-uniform electric field and has proven to be an ideal choice for the transport, aggregation, separation and characterization of particles at the nanoscale in microfluidic systems (38). To address the difficulty in separating exosomes in plasma, Ibsen and his team demonstrated that the alternating current electromotor (ACE) microarray device enables rapid isolation and recovery of glioblastoma exosomes from undiluted plasma (39). On this basis, Ayala-Mar and his colleagues further designed a miniature device called the DC-iDEP, which consists of a channel and two electrically insulated columns in order to generate different dielectric electrophoresis forces on the suspended objects (38). Notably, Tayebi’s group integrated acoustic radiation force (ARF) with dielectrophoresis and positioned a high-frequency inter-digital transducer (>10MHz) into the flow pathway, generating a force field based on both acoustic radiation and dielectrophoresis. Lateral translation of particles in the medium was observed as a result of the interplay of fluid drag force, ARF, and dielectrophoresis force fields, leading to the isolation of exosomes (40).

#### 3.1.4 Capture by functionalized microchannels

Modification of probe molecules on the inner surface of microfluidic channels is one of the most common methods for separation and capture of exosomes by immunoaffinity (41). Thus, exosomes in the body fluid are captured and accumulated in the device channel, while the rest of the body fluid is released. The formation of AuNC-exosome-AuR complexes was utilized by Yang and his group to develop a microfluidic device for the isolation of exosomes (42). This device was successful in extracting 5×10 (9) particles from a 5mL urine sample within 30 minutes. However, the separation of captured exosomes still faces a significant challenge due to the tough bond between the antigen and antibody. Kang’s team combined the OncoBean microfluidic device with antibodies to capture and release circulating exosomes, creating the ExoBean system. This system consists of both different immune mode layers that can be mechanically rotated to improve the binding probability between antibodies and exosomes, thus achieving specific isolation of exosomes (43).

However, it is also important to recognize that exosomes purification technologies are constantly developing, and conventional biomarkers may only distinguish the exosomes with specific cargoes. Therefore, with the adoption of new technologies, it will be necessary to refine some findings in the future.

### 3.2 Exosome detection based on microfluidics

Exosome detection methods overlap with respect to the exosome properties they examine. A flowchart summarizing exosome processing and analysis methods, then obtain more information about the advantages and disadvantages of techniques of interest from exosome detection section of **Figure 2**.

#### 3.2.1 Fluorescence detection

As we know, exosomes fixed in microfluidic chips could be specifically stained with fluorescent dyes (like PKH67, PKH26 and DiO) (44). Kanwar uses an “ExoChip”, staining exosomes with DiO and quantifying exosomes using a standard microplate meter (45). The combination of fluorescein and microfluidic technique also shows great potential for the detection of exosomes content. The Reversible Interaction Nanoscale Sorting Enrichment (RInSE) system utilizes the principle of inertial focusing in order to continuously isolate and analyze beads using flow cytometry. Exosomes were isolated using functional beads, and then introduced into the microchannel devised specifically to induce inertial focusing and facilitate buffer exchange. This method allowed for precise positioning of the beads, thus enabling continuous fluorescence analysis through flow cytometry (46).

#### 3.2.2 Electrochemical detection

In recent years, electrochemical detection has gradually developed into an effective way to detect exosome separation or accumulation, because electrochemical sensing devices can achieve high sensitivity and high efficiency detection through signal amplification (47, 48). Zhou’s group designed an electrochemical biosensor based on aptamers used for the quantification of exosomes with more objective and direct results. An aptamer specific to CD63 is fixed on the gold electrode surface, and the pre-labeled probe chain binds to the aptamer molecule fixed on the electrode surface. If exosomes are present, these beacons release the detection chain, resulting in a decrease in the electrochemical signal (49).

#### 3.2.3 Surface plasmon resonance detection

SPR is the resonance oscillation generated by the conduction electron at the interface of the particle’s negative conductivity and positive conductivity material excited by incident light (50, 51). SPR technology based on microfluidics has gradually come into view and become one of the main technical means in the field of exosome detection (52). Notably, Lee et al. described a method for Nanoplasma exosome (nPLEX) analysis as a new technique for quantification of exosomes. It works through the transmitted SPR of periodic nanopore arrays, in which each array is functionalized with antibodies for the analysis of various exosomal proteins (53).

#### 3.2.4 Single exosome detection

Intrinsic heterogeneity is one of the main factors hindering exosome analysis in body fluids. Single exosome detection may provide more accurate information of tumor progression. Because conventional flow cytometry is not capable of sensitively identifying nanosized exosomes, researchers have utilized aldehyde/sulfate latex beads to bind to the vesicles. These vesicles are then stained with fluorescent antibodies and analyzed for their protein markers. The percentage of positive beads, in which both the anti-GPC-1 antibody and Alexa-488-tagged secondary antibody were introduced, is defined as the percentage of GPC-1 positive exosomes (54). Recent study demonstrated a TIRF-based single-vesicle imaging assay was developed. This assay delivered molecular beacon probes into exosomes, resulting in an amplified fluorescence of the target microRNA. Through the direct visualization of individual vesicles and *in-situ* quantitative analysis of miR-21 in human serum samples, researchers discovered that this assay outperformed conventional polymerase chain reaction (PCR) assays in the diagnosis of cancer patients from healthy donor (55).

#### 3.2.5 Molecular beacons

MB is a probe that resembles a hairpin-shaped oligonucleotide. It is labeled with a fluorescent dye and a quencher at both ends. When MB hybridizes with the targeted sequence, it disrupts the hairpin structure and causes fluorescence to appear (56). MB-based biosensors were used to detect miR-375 and miR-574-3p in exosomes from human urine (57). As well as the expression level of miR-21, miR-375, and miR-27a were detected in exosomes from human serum (58). Furthermore, Lee et al. discovered that the hybridization of molecular beacons and miRNA-21 in exosomes from cancer cells and human serum produced strong fluorescent signals (59).

#### 3.2.6 Localized surface plasmon resonance

The occurrence of LSPR takes place when the frequency of the incident photon matches the overall vibration frequency of precious metal nanoparticles or metal conducting electrons. Joshi et al. devised a biosensor that utilizes LSPR for the label-free and nondestructive evaluation of exosomal miR-10b (60). In the subattomolar concentration range, this system displayed standard sensitivity to discriminate between miR-10b and miR-10a, despite having only a one-nucleotide difference. To accurately diagnose NSCLC, Wu et al. created an SPRi-based biosensor capable of detecting multiple exosomal miRNAs. They employed an Auon-Ag heterostructure and a DNA tetrahedral framework to amplify the SPR signal, enabling the identification of each exosomal miRNA with high sensitivity through different SPR signals (61).

## 4 Functional role of exosomes in GI cancer drug resistance

**Figure 3:**
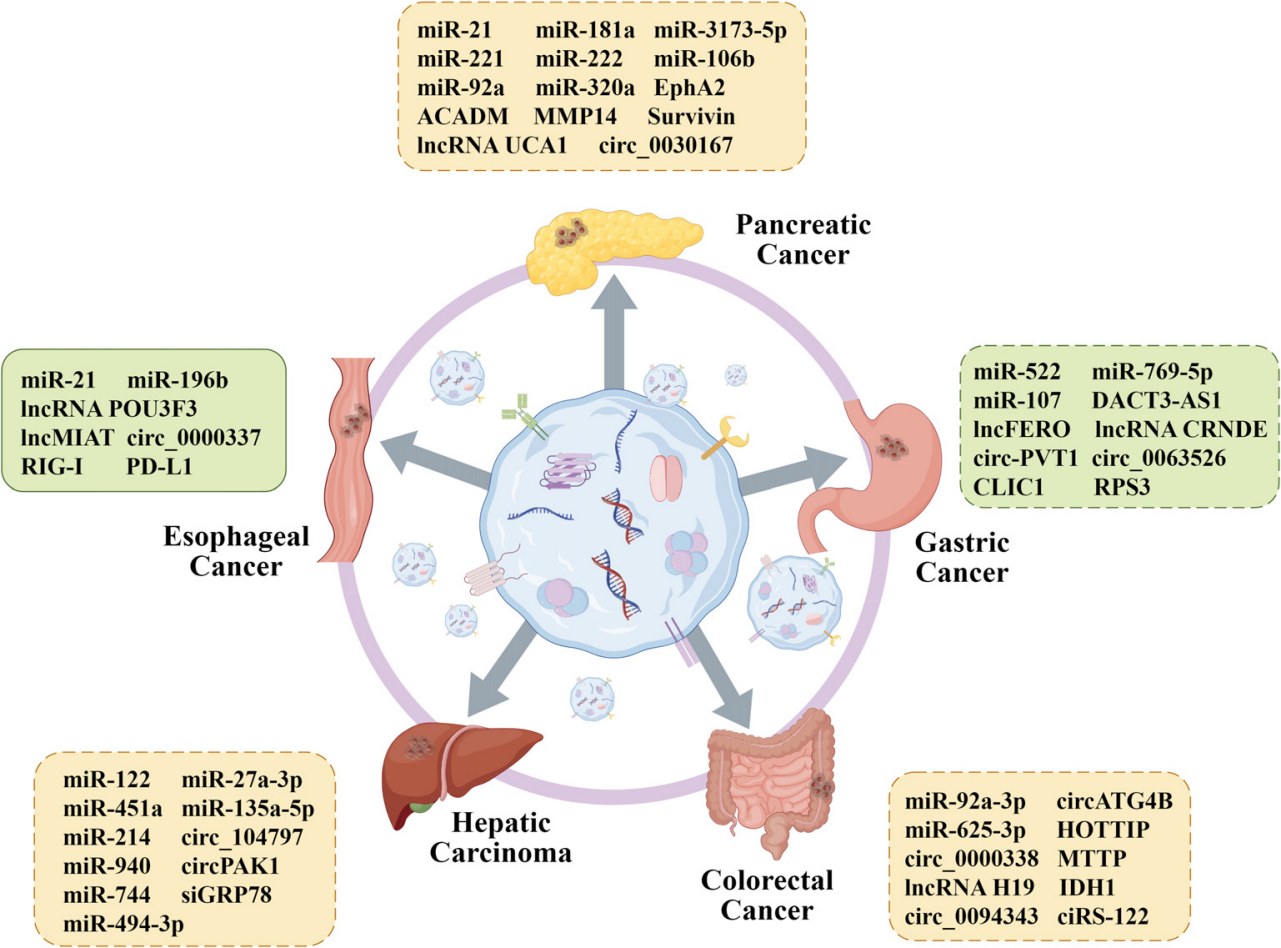
Functional role of exosome contents in GI cancer drug resistance. Exosomal differential proteins and nucleic acids from different cells play an important role in the reversal of drug resistance, providing favorable conditions for further promoting individualized treatment of GI cancer. By Figdraw.

In this article, GI primarily refer to malignant neoplasms arising from organs of the digestive system. Based on anatomical location, they are classified as EC, GC, PC, HCC, and CRC. From an aetiological perspective, environmental factors play different roles; for instance, helicobacter pylori infection is a key driver of GC, while hepatitis B virus/hepatitis C virus (HBV/HCV) infection and aflatoxin exposure are central to the pathogenesis of HCC (62, 63). Pathologically, EC can be divided into squamous cell carcinoma, which is associated with smoking and alcohol consumption and commonly seen in eastern Africa and eastern Asia, and adenocarcinoma, which often originates from Barrett’s esophagus (64, 65). GC is distinguished by the Lauren classification (intestinal, diffuse, and mixed types) and is frequently associated with mutations in genes such as tumor protein p53 (TP53), low-density lipoprotein receptor-related protein 1B (LRP1B), and AT-rich interaction domain 1A (ARID1A) (66). PC is dominated by Kirsten rat sarcoma viral oncogene homologue (KRAS) mutations (>90%), while CRC often exhibits characteristic gene changes in the adenomatous polyposis coli (APC)-KRAS-TP53 pathway (67, 68). Common mutations in HCC, such as those occurring in the telomerase reverse transcriptase (TERT), β-catenin 1 (CTNNB1), and TP53 genes, have also been identified (69). However, the specific mechanisms underlying the drug resistance of these five types of GI cancer remain unclear. Up to now, numerous research has confirmed that exosomes are tightly correlated to the occurrence and progression of drug resistance in GI cancer (**Figure 3**, **Table 1**). Therefore, in order to improve the outcomes of GI cancer patients, it is vital to investigate the specific exosomal molecule and signaling pathways related to drug resistance.

**Table 1:** Exosomal contents mediating the drug resistance of GI cancer.

Cancer type	Cell origin of exosome	Exosome content	Target(s)	Resistant type	Mechanism	Reference
Esophageal Cancer	CAFs	miR-21	PTEN	cisplatin	promoting the generation of M-MDSCs	Zhao et al. (70)
	tumor cells	miR-196b	EPHA7	paclitaxel and cisplatin	facilitating cell proliferation, invasion and EMT	Tan et al. (71)
	tumor cells	lncRNA POU3F3	———	cisplatin	inducing the transformation of NFs to CAFs	Tong et al. (72)
	tumor cells	MIAT	TAF1	PTX	increasing cell viability and inhibiting apoptosis	Zhang et al. (73)
	tumor cells	circ_0000337	JaK2	cisplatin	promoting cell growth and metastasis	Zang et al. (74)
	CAFs	RIG-I	IFN-β	cisplatin	enhancing cell proliferation and reducing apoptosis	Cui et al. (78)
	tumor cells	PD-L1	STAT3	PTX	increasing cell invasion and migration	Wang et al. (79)
Gastric Cancer	CAFs	miR-522	ALOX15	cisplatin and PTX	inhibiting ferroptosis in cancer cells	Zhang et al. (80)
	tumor cells	miR-769-5p	caspase 9	cisplatin	inhibiting apoptosis in cancer cells	Jing et al. (81)
	tumor cells	miR-107	HMGA2	5-FU and cisplatin	promoting the efflux of chemotherapeutic agents	Jiang et al. (82)
	CAFs	DACT3-AS1	miR-181a-5p	oxaliplatin	suppressing cell proliferation, migration, and invasion	Qu et al. (83)
	tumor cells	lncFERO	SCD1	cisplatin and paclitaxel	suppressing ferroptosis and enhancing stemness	Zhang et al. (84)
	M2-polarized macrophage	lncRNA CRNDE	PTEN	cisplatin	facilitating cell proliferation and inhibiting cell apoptosis	Xin et al. (85)
	tumor cells	circ_0063526	miR-449a	cisplatin	promoting cell proliferation	Yang et al. (88)
	tumor cells	circ-PVT1	miR-30a-5p	cisplatin	decreasing apoptosis and promoting invasion or autophagy	Yao et al. (89)
	tumor cells	CLIC1	——	vincristine	inhibiting apoptosis in cancer cells	Zhao et al. (91)
	tumor cells	RPS3	PI3K	cisplatin	decreasing cell apoptosis	Sun et al. (93)
Hepatic Carcinoma	CAFs	circZFR	STAT3	cisplatin	improving cell viability and proliferation, inhibiting apoptosis	Zhou et al. (95)
	M2 macrophage	miR-27a-3p	TXNIP	5-FU and cisplatin	promoting stemness, proliferation, migration and invasion of cancer cells	Li et al. (96)
	AMSC	miR-122	——	5-FU and sorafenib	enhancing cell apoptopsis and cell cycle arrest	Lou et al. (97)
	tumor cells	miR-135a-5p	VAMP2	DOX	promoting anti-apoptosis and cell proliferation	Wei et al. (98)
	hucMSCs	miR-451a	ADAM10	paclitaxel	restricting proliferation, migration, invasion and EMT of cancer cells	Xu et al. (103)
	tumor cells	miR-214	P-gp and SF3B3	oxaliplatin	reducing cancer cell viability and invasion	Semaan et al. (104)
Pancreatic Cancer	CAFs	miR-3173-5p	ACSL4	GEM	inhibiting ferroptosis	Qi et al. (115)
	CAFs	miR-106b	TP53INP1	GEM	promoting cell viability and proliferation	Fang et al. (116)
	CAFs	miRNA-320a	PTEN	GEM	facilitating macrophage M2 polarization, cancer cell proliferation and invasion	Zhao et al. (117)
	PSCs	lncRNA UCA1	EZH2	GEM	inhibiting apoptosis in cancer cells	Chi et al. (118)
	tumor cells	ACADM	GPX4	GEM	inhibiting ferroptosis in cancer cells	Yang et al. (121)
	tumor cells	EphA2	——	GEM	——	Fan et al. (122)
	tumor cells	MMP14	CD44	GEM	promoting colony formation of GEM-sensitive cells	Li et al. (123)
	KRAS-mutant tumor cells	Survivin	——	paclitaxel	promoting cell survival and inhibiting apoptotic	Chang et al. (125)
Colorectal Cancer	CAFs	miR-92a-3p	FBXW7	5-FU/oxaliplatin	enhancing stemness and EMT	Hu et al. (126)
	CAFs	lncRNA H19	miR-41	oxaliplatin	promoting the stemness of CSCs	Ren et al. (127)
	CAFs	miR-625-3p	CELF2	oxaliplatin	promoting EMT and reducing apoptosis in CRC cells	Zhang et al. (128)
	tumor cells	P-STAT3	——	5-FU	inhibiting apoptosis in cancer cells	Zhang et al. (129)
	tumor cells	HOTTIP	miR-214	mitomycin	decreasing apoptosis and DNA damage of CRC cells	Chen et al. (130)
	tumor cells	circ_0000338	miR-217 and miR-485-3p	5-FU	promoting cell proliferation and decreasing cell apoptosis	Zhao et al. (131)
	adipocyte	MTTP	PRAP1	oxaliplatin	suppressing ferroptosis	Zhang et al. (132)
	tumor cells	circATG4B	circATG4B‐222aa	oxaliplatin	enhancing autophagy	Li et al. (136)
	tumor cells	ciRS-122	PKM2	oxaliplatin	enhancing glycolysis	Wang et al. (137)
	tumor cells	circ_0094343	miR-766-5p	5-FU,oxaliplatin and DOX	inhibiting cell proliferation, clone formation and glycolysis	Li et al. (138)
	tumor cells	IDH1	NADPH	5-FU	promoting proliferation and glycometabolism of cancer cells	Yang et al. (139)

### 4.1 Esophageal cancer

EC, a common and aggressive disease, is divided into esophageal squamous cell carcinoma (ESCC) and esophageal adenocarcinoma (EAC). Numerous exosomal non-coding RNAs and proteins may have significant roles in EC drug resistance, according to earlier research. For instance, Zhao et al. discovered that in ESCC patients, monocytic myeloid-derived suppressor cells (M-MDSCs) are associated with susceptibility to cisplatin (70). Further mechanism experiments revealed that the exosomal miR-21 inhibits PTEN to activate the signal transducing activator of transcription 3 (STAT3) signaling, which converts monocytes into M-MDSCs and eventually promotes cisplatin resistance. To explore the mechanisms of activation of CAFs by normal fibroblasts (NFs), Tong et al. conducted a series of experiments and proved that exosomal lncRNA POU3F3 secreted by tumors drives NF differentiation into CAFs and the development of cisplatin resistance in ESCC cells (72). Meanwhile, they proposed that POU3F3 has the potential to be an independent prognostic biomarker for predicting cisplatin resistance in ESCC. Another study discovered that the transport of circ_0000337 by exosomes could promote EC cell cisplatin resistance, as the circ_0000337 functioned as a miR-377-3p ceRNA to raise JaK2 expression (74). Exosome has also been shown to transfer miR-130a-3p from cisplatin-resistant EC cells to sensitive cells, thereby enhancing resistance by regulating ferroptosis (75).

Compared with the nucleic acids, there are relatively few studies on exosomal proteins related to EC chemotherapy resistance. The cytoplasmic pattern recognition receptor retinoic acid-inducible gene-I (RIG-I) is responsible for the host’s identification of RNA virus infection and the subsequent stimulation of type I interferon (IFN) production in innate immune cells (76). RIG-I may be a target for cancer treatment since it has been demonstrated to control radiotherapy by interfering with the creation of DNA repair proteins (77). Cui et al. indicated that exosomes from CAFs carry RIG-I to activate the RIG-I/IFN-β signaling pathway, which promotes cell expansion and drug resistance (78). Notably, exosomal PD-L1 has also been proven to increase paclitaxel (PTX) resistance in ESCC cells by modulating the STAT3/miR-21/PTEN/Akt signaling pathway, possibly providing a novel perspective on the strategy selection for immune treatment (79).

### 4.2 Gastric cancer

The preferred course of action for patients with unresectable GC is chemotherapy, and one of the main obstacles to improving the prognosis of these patients is drug resistance. A recent study showed that miR-522, sorted into exosomes secreted from CAFs, suppress ALOX15 expression and eventually decreased sensitivity to cisplatin and PTX (80). The *in vivo* study also showed that knockdown of miR-522 in CAFs observably suppress tumor growth in mice. Moreover, Jing et al. found that exosomal miR-769-5p targets the downstream caspase pathway of apoptotic signaling molecule caspase-9 inactivation and mediates the degradation of p53 protein through the E3 ubiquitin ligase NEDD4L, ultimately inhibiting cell apoptosis and promoting cisplatin resistance (81). Exosome has also been proven to transfer lncRNA DACT3-AS1 from CAFs to GC cells, which sensitizes GC cells to oxaliplatin therapy by combining the impacts of ferroptosis and apoptosis (83). More importantly, immune cells in the TME could also regulate GC drug resistance. M2-polarized macrophages could release exosomal lncRNA CRNDE to GC cells to induce cisplatin resistance by decreasing PTEN expression (85). Meanwhile, Zhou et al. discovered that miR-194 also plays the same role in this process (86). The circ50547 in exosomes can inhibit the oxaliplatin-induced apoptosis, and mechanistic studies revealed the miR-217/HNF1B signaling pathway is involved in this process (87). Yang et al. initially postulated that exosomal circ_0063526 could induce cisplatin resistance in GC cells by sponging miR-449a to mediate SHMT2 expression, and finally proved the hypothesis (88). Exosome-derived circ-PVT1 has been reportedly related to cisplatin resistance of GC, and si-circ-PVT1 transfection increase cisplatin chemosensitivity through the miR-30a-5p/YAP1 axis (89).

In spite of RNAs, exosome-specific proteins also take part in mediating the process of drug resistance in GC. Researchers have indicated that the protein CLIC1 could insert into lipid membranes to form a chloride ion channel in response to stimuli, which has been linked to the resistance of various human cancers (90, 91). Song et al. conducted a series of experiments *in vitro* and *in vivo* in order to explore the specific mechanism by which β-elemene exerts its role in reversing cisplatin resistance in GC (92). Their findings, as reported in the literature, demonstrate that β-elemene is able to inhibit tumor growth and chemotherapy resistance by targeting METTL3 in exosomes. Sun et al. established a cisplatin-resistant gastric cancer cell line, SGC7901R, and analyzed the connection between its exosomal proteins and the resistance of GC (93). They figured out that the RPS3, the most significantly different exosomal protein, blocks the inhibition of cisplatin on GC cells by the PI3K-Akt-cofilin-1 axis. Hence, the cargoes of exosomes affect chemotherapy resistance of GC and have the potential to be a new strategy for cancer treatments.

### 4.3 Hepatocellular carcinoma

HCC, a common type of liver cancer, mostly develops in patients with cirrhosis. Although systemic chemotherapies are recommended for advanced patients, chemoresistance influences therapeutic effect and makes advanced HCC patients face grim challenge (94). Researchers have already focused on exosomal cargos to reduce drug resistance. CircZFR is elevated in CAFs and co-culture with CAF-derived exosomes could remarkably increase cell viability even after treating with cisplatin. Researchers further revealed that knockdown of circZFR suppress cell proliferation and accelerate apoptosis (95). Li et al. figured out that M2 macrophage-exosomes reduce the inhibition rate of 5-fluorouracil (5-FU) on cancer cell proliferation by transporting miR-27a-3p to HCC cells (96). Another study found that adipose tissue-derived mesenchymal stem cells (AMSC) could efficiently package miR-122-expressed plasmids into secreted exosomes (97). These exosomes increase cell apoptosis and cycle arrest, enhancing HCC cell chemosensitivity to 5-FU and sorafenib. As one of the most widely prescribed clinical anticancer agents, doxorubicin hydrochloride (DOX) resistance also seriously affects the therapeutic effect. For instance, exosomal miR-135a-5p inhibits the DOX-induced apoptosis, and mechanistic studies revealed that miR-135a-5p targets vesicle-associated membrane protein 2 (VAMP2) to function (98). Exosome-delivered miR-425-5p enhances the expansion of regulatory T cells (Treg) and promotes immune evasion of HCC cells that drive resistance to oxaliplatin and capecitabine (99). This was also confirmed by vivo studies in mouse xenograft models. It has also been experimentally demonstrated that exosome cargoes are associated with resistance to targeted drugs or immunotherapy in HCC (100–102). It has been shown that sorafenib and lenvatinib are tyrosine kinase inhibitors, both belonging to the first-line systemic therapeutic drugs (105). Several studies have shown that circ_104797, circPAK1, miR-940, miR-494-3p, miR-744, and siGRP78 are all transmitted by exosomes and involved in resistance to sorafenib and Lenvatinib (106–111). Collectively, these results suggest that exosomes might provide potential treatment targets for HCC in addition to being promising prognostic indicators.

### 4.4 Pancreatic cancer

PC is among the worst malignancies. Only up to 20% of patients have a possibility of surgical resection since the early signs are mainly concealed. Nevertheless, local recurrence or metastasis still occurs in over 80% of patients who undergo successful surgery (112). As a result, chemotherapy with or without targeted therapies is typically recommended as the primary treatment for unresectable PC (113). However, drug resistance must be addressed immediately as acquired resistance to these drugs is common. CAFs, the predominant component in the TME, participate in drug resistance process of PC by secreting exosomes. It was reported that exosomal miRNAs from pancreatic CAFs are significantly different from the gemcitabine (GEM)-treated CAFs, as miR-92a, miR-21, miR-181a, miR-221, and miR-222 are upregulated in the latter (114). These miRNAs promote GEM chemoresistance by modulating PTEN in pancreatic ductal adenocarcinoma (PDAC). Qi et al. suggested that miR-3173-5p in exosomes secreted from CAFs inhibits ferroptosis by inhibiting ACSL4 and subsequently promotes GEM resistance in cancer cells (115). Silvie et al. demonstrated that exosomal miRNA-320a is transmitted from CAFs into macrophages to facilitate its M2 polarization through activating PTEN/PI3Kγ pathway (117). MiRNA-320a overexpression results in PC cell migration and invasion. Apart from CAFs, it was reported that exosomes from hypoxic pancreatic stellate cells (PSCs) augment malignant phenotypes and chemoresistance. Subsequently, it was shown that lncRNA UCA1 is significantly expressed in hypoxic PSC-derived exosomes and induces the resistance to GEM in PANC-1 cells (118). The PC stem cells have been shown to release exosomes, and the miR-210 within the exosomes can modulate the M2 polarization of macrophages to reduce GEM sensitivity of PC cells (119). Noda et al. suggested that miR-199a-3p expression in exosomes secreted by PC-associated adipocytes was markedly increased, and the miR-199a-3p was transferred to PC cells, which was related to GEM resistance and poor prognosis (120).

Exosomal protein transport also has an impact on the chemoresistance of PC cells. Medium-chain acyl-CoA dehydrogenase could upregulate glutathione peroxidase 4 (GPX4) and inhibit ferroptosis to promote GEM resistance (121). EphA2 has been proven to mediate GEM resistance in sensitive PC cells (122). Additionally, the exosome-transferred matrix metalloproteinase 14 (MMP14) enhance the accumulation of CD44 protein and promote colony formation of GEM-sensitive cells (123). Wang et al. found that down-regulation of PPP3CB could inhibit the growth and promote the death of PC cells, thereby suppressing their resistance to GEM (124). Survivin could be found in exosomes from the serum of KRAS-mutant PDAC patients. When PDAC cells are treated with Survivin-loaded exosomes, the efficacy of PTX-mediated cell death is significantly compromised, and Survivin inhibitors could reverse the impact (125). Notably, these studies present novel approaches for investigating the mechanism of conquering drug resistance in PDAC by exosomes.

### 4.5 Colorectal cancer

As a widespread clinical disease, chemotherapy-resistant CRC has been an insurmountable problem in recent years. Statistical data show that chemoresistance of CRC can be related to epithelial-mesenchymal transition (EMT) and cancer stem cells (CSCs). Prior research has demonstrated that CAF-exosome miR-92a-3p enhances stemness and EMT phenotype by targeting FBXW7 and MOAP1, thus promoting chemoresistance to 5-FU/oxaliplatin in CRC cells (126). Exosome-enriched lncRNA H19 has also been shown to increase CSC stemness and oxaliplatin resistance by sponging miR-41 to activate the Wnt/β-catenin axis (127). Furthermore, Zhang et al. pointed out that exosomal miR-625-3p from CAFs also promotes EMT and reduces apoptosis in CRC cells (128). Apoptosis is the most studied modality of regulated cell death associated with drug resistance in CRC. Phosphorylate-STAT3 delivered by exosomes from resistant cells may decrease apoptosis and thereby increase 5-FU resistance in recipient cells by reducing caspase cascade activation (129). Further mechanism study showed that MTTP, transmitted by exosomes, targets the MTTP/PRAP1/ZEB1 axis and decreases PUFA levels to suppress the ferroptosis of malignant cells (132). To investigate the mechanism of 5-FU resistance in CRC, Chen et al. detected RNA and protein in exosomes and confirmed miR-149-5p could sensitize resistant cells. In addition, miR-224-5p from tumor exosomes and circ_0067557 from CAF exosomes have also been considered to play roles in this process (133).

Autophagy is a self-degrading process that may play a dual role in chemoresistance. It can be a direct killer of cancer cells, but also shield them from the effects of chemotherapeutic agents. The latter is why tumor cells develop drug resistance through autophagy (134). Pan et al. suggested that oxaliplatin-resistant CRC cells secreted exosomes, by which circATG4B could be transported to enhance autophagy and subsequently induce oxaliplatin resistance in sensitive cells (135). The link between metabolic reprogramming within cancer cells and the development of chemotherapy resistance has been demonstrated. Increased glucose uptake and aerobic glycolysis promote CRC chemoresistance, in which several glycolytic enzymes have been implicated (136). Oxaliplatin resistant cell-derived exosomes could be endocytosed into the sensitive cells, and exosomal ciRS-122 enhances accumulation of glycolysis and promotes tumor progression to induce oxaliplatin resistance by promoting PKM2 expression (137). IDH1 expression in 5-FU-resistant exosomes was markedly higher than that in sensitive exosomes, and exosomes with elevated IDH1 increased the intracellular NADPH levels, enhancing the chemoresistance of CRC cells (139). Tong et al. conducted a series of experiments and proved that exosomal FOSL1 secreted by CAFs promotes oxaliplatin resistance in CRC cells (140). Furthermore, several other mechanisms by which exosome contents regulate drug resistance in CRC remain unmentioned, such as angiogenesis, drug efflux pump transfer, and DNA methylation.

In summary, the above results highlight the crucial roles of exosomal cargos in the regulation of drug resistance in tumors, whether by directly interacting with drugs or mediating tumor cell survival.

## 5 Potential clinical applications of exosomes in GI cancer drug resistance

In the previous sections, we discussed the influence of exosomes on the occurrence and development of chemotherapy resistance in GI cancer. These observations may contribute to enhancing sensitivity and efficacy of chemotherapeutic drugs, which has significant potential for clinical application. Based on this, we will proceed to examine the clinical value of exosomes in drug resistance of GI cancer, which can be categorized into two main areas: as biomarkers and as therapeutic tools.

### 5.1 Exosomes as biomarkers for predicting drug resistance of GI cancer

Exosomes carry tremendous information about an individual’s tumor condition, making them a new tool for early diagnosis and prognostic tracking. Compared with conventional biomarkers, exosomes have more benefits in liquid biopsy: easier extraction due to their abundance in body fluids, higher representation due to the secretion by living cells and greater stability due to the lipid bilayers (141). Up to now, a multitude of evidences have shown that exosomes could be reliable biomarkers for tumor screening and diagnosis, several of which are currently in clinical trials (**Table 2**).

**Table 2:** Exosomal contents as candidate biomarkers in the prognosis of GI cancer.

Exosomal biomarker	Cancer type	Sample type	Source	Clinical sample size	Ref
TNFRSF10B and ILF3	ESCC	tumor tissues	CAFs	TP N = 63	wang et al. (143)
GPC3	GEA	Serum	tumor cells	TP N = 49,HC N = 56	Rahbari et al. (144)
CD63	GC	tumor tissues	tumor cells	TP N = 595	Miki et al. (145)
CD133	PC	ascites	tumor cells	TP N = 19	Sakaue et al. (146)
Rab27b	GC	tumor tissues	tumor cells	TP N = 178	Nambara et al. (148)
PPP5C	PDAC	tumor tissues	tumor cells	TP N = 178	Fu et al. (150)
DPP4	CRC	tumor tissues	tumor cells	TP N = 40	Zheng et al. (151)
miR-125b	CRC	plasma	tumor cells	TP N = 55	Yagi et al. (152)
miR-451a	CRC	tumor tissues	hucMSCs	TP N = 92	Xu et al. (103)
miR-196b-5p	CRC	tumor tissues	tumor cells	TP N = 90	Ren et al. (153)
MiR-155	PDAC	tumor tissues	tumor cells	TP N = 45	Mikamori et al. (154)
miR-21	PC	serum	tumor cells	TP N = 32,HC N = 29	Goto et al. (156)
miR-500a-3p	GC	plasma	tumor cells	TP N = 55	Lin et al. (157)
miR‐769‐5p	GC	tumor tissues	tumor cells	TP N = 75	Jing et al. (81)
lncCACC	CRC	plasma	tumor cells	TP N = 59	Zhang et al. (159)
DACT3-AS1	GC	tumor tissues	CAFs	TP N = 93	Qu et al. (83)
lncRNA PVT1	HCC	serum	tumor cells	TP N = 40	Lai et al. (160)
lncRNA-GC1	GC	plasma	tumor cells	TP N = 375	Song et al. (161)
hsa-circRNA-G004213	HCC	plasma	tumor cells	TP N = 50	Qin et al. (164)
circHIPK3	GC	Serum	tumor cells	TP N = 20	Shang et al. (165)
circ_0063526	GC	plasma	tumor cells	TP N = 97	Lu et al. (166)
circUHRF1	HCC	plasma	tumor cells	TP N = 240	Zhang et al. (101)

#### 5.1.1 Exosomal protein

Exosomal proteins, whether on the surface or within the membrane, provide abundant, stable, sensitive, and distinct information (142). Multitude evidence revealed that the proteins may be able to monitor progression of GI cancer. Wang et al. established a risk model with TNFRSF10B and ILF3 for ESCC, and the risk scores were positively correlated with the IC50 of multiple chemotherapeutic drugs by spearman analysis (143). As an oncofetal protein, exosomal glypican 3 (GPC3) is a predictive biomarker for primary gastro-esophageal adenocarcinoma (GEA), as patients with GEA had lower overall survival (OS) when the GPC3 levels were higher (144). In stage III and stage IV GC, patients with exosomal surface marker CD63 expression had significantly lower OS rates than those with CD63-negative expression (P < 0.0001 and P = 0.0063, respectively) (145). In addition, it has been proven that highly glycosylated CD133 in ascites-derived exosomes may serve as a prognostic biomarker for advanced PC (146). Members of Rab GTPases Rab27a and Rab27b control exosome release (147). Nambara et al. demonstrated that Rab27b knockdown could suppress peritoneal metastasis in the xenograft mouse model of GC, and they proved the correlation between elevated Rab27b and poor OS rates in 178 GC patients from the local hospital and 406 from the TCGA database (148). Liang et al. established a predictive model for CRC chemoresistance with EXOC2, EXOC3 and STX4 in exosomes, and the area under curve (AUC) of 5-year progression-free survival (PFS) was 0.804 (149). Another research group also found significant differences in the level of dipeptidyl peptidase IV (DPP4) in exosomes from 5-FU resistant and sensitive cells (151). After analysis of the small sample of clinical data, researchers pointed out the higher expression of DPP4, and the lower differentiation degree of colon cancer tissues, which predicted a poor prognosis.

#### 5.1.2 Exosomal miRNAs

Differential expression of miRNA in exosomes can serve as biomarkers to detect treatment responses for GI cancer. Yagi et al. compared miR-125b level in plasma exosomes between CRC clients with progressive and stable disease receiving FOLFOX-based first-line chemotherapy and discovered miR-125b was upregulated in patients with progressive disease (152). In addition, COX multivariate analysis revealed miR-125b as an independent predictor for PFS (P = 0.041). Hence, exosomal miR-125b might be a useful minimally invasive indicator for CRC chemoresistance. Xu et al. discovered that patients with HCC who were in the low or high miR-451a expression groups had significantly different survival times (P<0.05), suggesting that patients with lower expression levels of miR-451a had a worse prognosis (102). Furthermore, miR-196b-5p was highly enriched in serum exosomes of CRC patients, which promoted stemness and chemoresistance of CRC cells (153). Notably, OS was considerably lower in individuals with high than in low miR-196b-5p levels (P = 0.006). MiR-155 was identified as a clinical resistance predictor (154). High expression of miR-155 was found in the exosome generated from GEM-resistant cell lines, and PDAC patients with high expression of miR-155 had shorter disease-free survival time (P = 0.021) and OS time (P = 0.008) than PDAC patients with low expression. The studies have indicated that miR-454-3p, miR-7974, miR-3615, miR-130b-3p and miR-4326 can collectively predict the efficacy of oxaliplatin in treating CRC (AUC = 1). It is anticipated that these will become biomarkers for predicting treatment resistance (155). Additionally, ROC curve analysis indicated that the miR-500a-3p levels in plasma exosomes could differentiate between cisplatin resistant and sensitive group in stage III GC patients (AUC = 0.843), and miR-500a-3p overexpression suggested a dismal prognosis (157). In a clinical trial (NCT06388967), the researchers plan to test the previously identified combination of 13 exosomal miRNAs in a larger international cohort study to confirm the effectiveness of this method in accurately identifying PDAC at its early stages (158).

#### 5.1.3 Exosomal lncRNAs and circRNAs

Several recent studies suggest that exosomal lncRNAs and circRNAs could be molecular markers for monitoring tumor progression. LncCACC was upregulated and enhanced CRC resistance to chemotherapy (159). Its expression in peripheral plasma exosomes of CRC patients could predict the chemotherapy effect. DACT3-AS1, transferred by exosomes, was decreased in GC tissues, which reduced oxaliplatin sensitivity and resulted in poor prognosis of GC patients (AUC = 0.6940) (83). Song et al. found that in GC patients receiving adjuvant chemotherapy, high levels of exosomal lncRNA-GC1 expression often indicated significantly shorter survival (P < 0.05) (161). In the clinical trial NCT05334849, the levels of lncRNA-GC1 in circulating exosomes were also measured to monitor and predict the response of GC patients to immunotherapy, and the expected results were shown (162). Transarterial chemoembolization (TACE) has been recommended by most clinical practice guidelines as standard treatment for patients with intermediate stage HCC due to its survival benefits (163). According to reverse transcription quantitative polymerase chain reaction (RT-qPCR) results, hsa-circRNA-G004213 was markedly increased after TACE (P < 0.01) (164). And researchers also demonstrated that exosomal hsa-circRNA-G004213 promoted the cisplatin sensitivity of HCC cells and was related to prognosis of liver cancer patients following TACE, which might be a biomarker for monitoring TACE effect. Recently, Shang et al. demonstrated that circHIPK3 was expressed more in the serum exosomes of GC patients who were resistant to cisplatin, indicating that exosomal circHIPK3 may be a biomarker for the clinical effectiveness of cisplatin chemotherapy (165). The aforementioned studies have partially validated the involvement of exosomes-based liquid biopsy in tumor resistance; nevertheless, additional samples and trials are required to validate their clinical value in tumor diagnosis and treatment.

### 5.2 Exosomes as therapeutic tools for drug resistant GI cancer treatment

**Figure 4:**
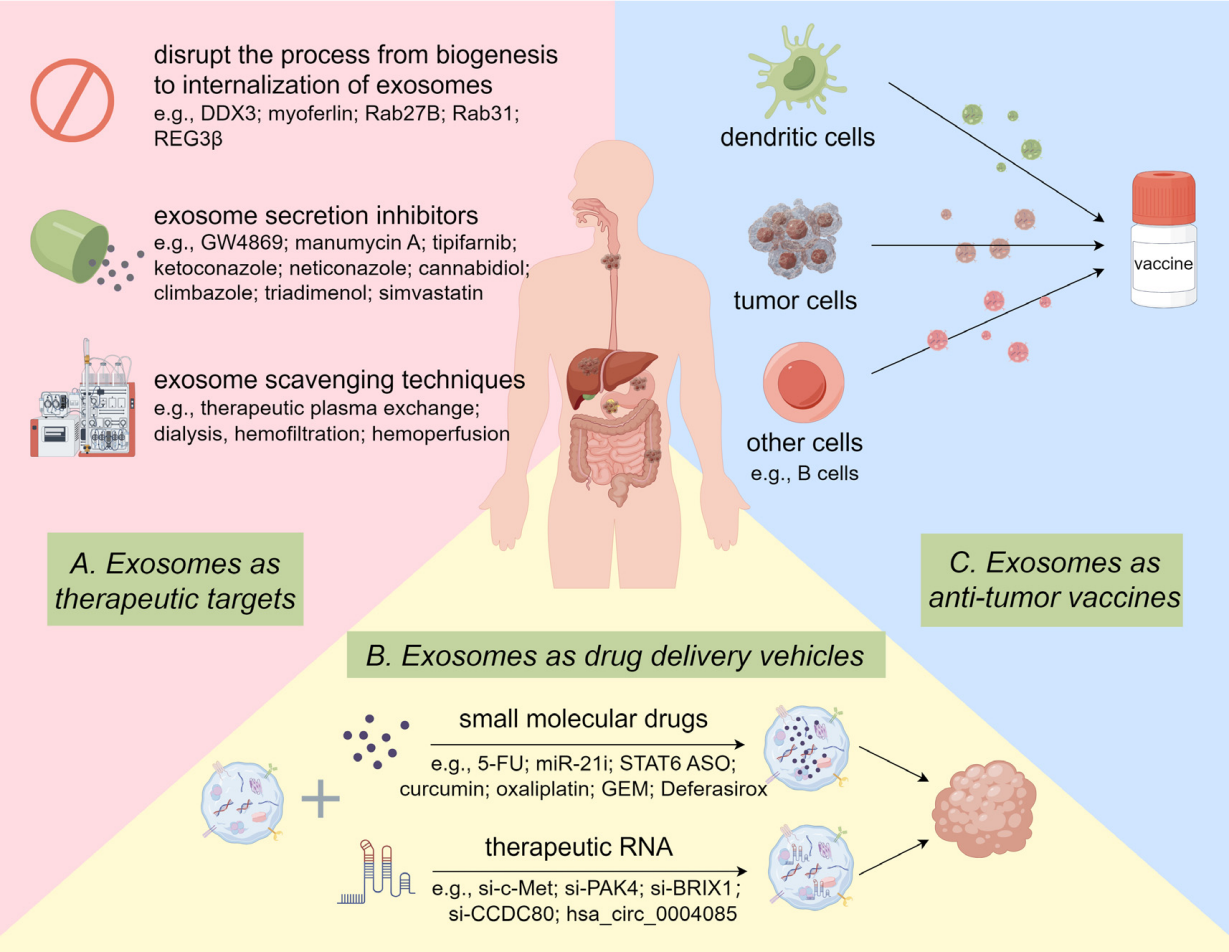
Potential Clinical Applications of Exosomes in GI Cancer drug resistance. Targeted inhibition of oncogenic exosomes through gene editing and pharmacological inhibitors and scavenging techniques. Delivery of therapeutic RNA or small molecule drugs to tumor cells via exosomes. Exosomes from various cells function as vaccines to activate anti-tumor immune responses. By Figdraw.

In addition to monitoring the efficacy of tumor chemotherapy as prognostic markers, exosomes were also employed to enhance drug effectiveness through a variety of pathways for therapeutic purposes. Currently, the exploitation and design of therapeutic exosomes center around the following three main aspects. On the one hand, “Natural Nanomedicine” uses exosomes to transfer molecular drugs by exosomes for cancer treatment. On the other hand, “Therapeutic Target”, namely specific blockade of cancer-promoting exosome transport to suppress tumor development. Finally, “Anticancer Vaccine” refers to vaccination with certain specific exosomes to activate the anti-tumor response of immune cells (**Figure 4**).

#### 5.2.1 Exosomes as drug delivery vehicles

As an endogenous bio-vehicle, exosomes have many advantages compared with conventional drug delivery systems such as synthetic polymers, liposomes, micelles, super magnetic particles, proteins, and recombinant viral vectors (167). It has been mentioned previously that exosomes are present in the majority of bodily fluids. Numerous edible plants have been shown to secrete exosomes, which create conditions for their abundant production (168). Further, exosomes are structurally stable and their membranes can protect the contents from degradation in harsh conditions. Notably, exosomes are also relatively non-immunogenic and non-toxic because of their similar composition to the body’s cells (169). Another benefit of exosomes is that they can deliver small molecular drugs or siRNAs across the blood-brain barrier, which is impossible with most nanocarriers (170). In addition, it is easier to modify drug carrying ability and tissue targeting specificity of exosomes (171). Therefore, exosomes deserve to be considered as potential vehicles for the delivery of small molecule drugs to treat cancer.

The delivery of therapeutic RNA, specifically siRNA, by exosomes to tumor cells is currently a popular area of cancer research. As the basis for cancer metastasis, c-Met is essential for tumor proliferation, invasion, and metastasis. Although the exact molecular mechanism is still unclear, Zhang et al. constructed si-c-Met loaded by exosomes and delivered it into GC cells, reversing the resistance to cisplatin in GC (172). Huang et al. loaded siRNAs targeting coiled-coil domain-containing protein 80 (CCDC80) into exosomes and delivered them to the CRC liver metastasis mouse model, which prolonged the overall survival of the mice (173). P21-activated kinase 4 (PAK4) was found to be oncogenic, and the encapsulating si-PAK4 in PANC-1 cell-derived exosomes successfully suppressed pancreatic tumors *in vitro* and *in vivo* (174). Yu et al. developed engineered exosomes carrying si-BRIX1, which could induce nucleolar stress and thereby enhance the efficacy of 5-FU against CRC (175). Studies have also shown that hsa_circ_0004085 could be packaged into exosomes, which effectively inhibited ER stress in the recipient cells and enhanced their resistance to oxaliplatin and 5-FU (176).

Liang et al. found an example of exosomes delivering anticancer drugs to overcome chemoresistance in CRC (177). They generated target-specific exosomes by engineered 293T cells and packaged 5-FU and miR-21 inhibitor oligonucleotide (miR-21i) into the exosomes. The engineered exosome delivery system markedly enhanced cytotoxicity and improved the effect of cancer treatment. CDK-004, consisting of exosomes carrying a STAT6 anti-sense oligonucleotide (ASO), was first introduced in a multi-center clinical trial in advanced HCC, and liver metastases from GC or CRC in 2023 (NCT05375604) (178). It was administered intravenously as a single agent and inhibited tumor development by repolarizing M2 macrophages. Although the clinical trial had to be terminated due to the company bankruptcy, it provided ideas for early clinical entry of exosome-based drugs in oncology. Research (NCT01294072) into the delivery of curcumin to colon tumor cells via plant-secreted exosomes to enhance its anti-tumor activity has also entered phase I clinical trials (179). The exosome-encapsulated 5-FU has also been proven to enhance CRC cell nuclear fragmentation and apoptosis in comparison to the exact same dose of free 5-FU (180). Wang et al. synthesized a novel modified exosome loaded with oxaliplatin, which could enhance the chemosensitivity of CRC by regulating mitochondrial function. The validity of these findings has been substantiated through *in vitro* experimentation and in orthotopic cancer models (181). Furthermore, M1 macrophage-derived exosomes loaded with GEM and Deferasirox could reduce cell viability and adhesion ability of GEM-resistant PC cells (182).

#### 5.2.2 Exosomes as therapeutic targets

As previously mentioned, exosomes mediate intercellular communication, and tumor-derived exosomes (TDEs) play an essential part in tumorigenesis and development. Thus, it is feasible for targeted inhibition of exosomes, especially TDEs, to improve the efficacy of cancer therapy. A clinical trial (NCT02393703) has already been conducted to investigate whether exosome activity is associated with disease recurrence and outcomes in PC patients (183). Theoretically, disrupting any step in the process from their biogenesis in tumor cells to internalization by recipient cells can successfully result in TDE dysregulation, such as inhibiting TDE biogenesis and packaging, modulating TDE trafficking, and blocking TDE internalization (184). Up to now, genetic manipulation and pharmacological inhibition are the most researched methods for targeting the inhibition of TDEs (185). DDX3, a DEAD-box RNA helicase, was reported to suppress the release of HCC exosomes, which contributes to its anti-tumor property (186). He et al. proposed that targeting vesicle trafficking-related protein myoferlin could be a promising opportunity to treat CRC clinically, as myoferlin suppressed exosome secretion and internalization and thereby reduced the invasive capacity of tumor cells (187). Li and his team found Rab27B was overexpressed and reduced drug concentration in 5-FU resistant HCC cells by encouraging exosome release, while genetic knockdown of Rab27B restored sensitivity to chemotherapy drugs and improved their therapeutic effects (188). Recent studies have suggested that Rab31 also played the same roles in GC development and could be a possible target for therapeutic interventions (189). Besides, regenerating islet-derived protein 3 beta (REG3β) was proved to block the capture of TDEs by target cells, thereby reversing the malignant phenotype of PDAC cells (190).

Pharmacological inhibition is another form of targeting TDEs for tumor therapy. For instance, as a blocker of nSMase2, GW4869 has been applied as an exosome secretion inhibitor to regulate tumor progression in several experiments. Wang et al. injected GW4869 directly into the CRC tumor, which suppressed TDEs secretion and tumor growth (191). In addition, Richards discovered that GW4869 negatively affected exosome secretion from CAFs, resulting in the re-sensitization of PDAC cells to gemcitabine (192). M2 macrophage-derived exosomes were demonstrated to promote migration of GC cells, and GW4869 treatment significantly reduced the exosome-induced lung colonization in mice models of GC (193). In rat models, pantoprazole reduced the quantity of exosomes in the liver and serum, indicating the potential to reduce liver tumorigenesis (194). Additionally, there are many other compounds, like manumycin A, cannabidiol, tipifarnib, ketoconazole, neticonazole, climbazole, triadimenol, and simvastatin, were demonstrated to affect the exosome release (195, 196). Several direct exosome scavenging techniques have also been investigated, such as therapeutic plasma exchange, dialysis, hemofiltration, and hemoperfusion. However, the above treatments carry an increased risk of trauma and infection, which is an additional burden for cancer patients.

#### 5.2.3 Exosomes as anti-tumor vaccines

It has been reported that all clinically apparent tumors have evolved mechanisms of immune evasion, which are achieved by altering tumor cell self-modification and the tumor immune microenvironment (TIME) (197). Given these mechanisms, researchers have proposed several immunotherapies applied in cancer treatment, such as oncolytic virus therapies, cytokine therapies, adoptive cell transfer, immune checkpoint inhibitors, and cancer vaccines (198). Despite their clear efficacy, these therapies still have their own limitations and side effects. Exosome-based immunotherapy is considered to be a potential treatment option for GI cancer that overcomes the toxicity and instability of traditional tumor vaccines (199). Dendritic cells (DCs), professional antigen-presenting cells, can stimulate tumor antigen-specific T cells to trigger anti-tumor immune response (200). On the basis of maintaining the immunostimulatory function of DCs, DC-derived exosomes (DEXs) also show their stability, controllability, and low immunosuppression as anticancer vaccines (201). Wang et al. suggested that DEXs treated with hyperthermic CO2 reduced the expression of HSP70 and enhanced its own immune effect (202). Although definitive *in vivo* studies have not yet been performed, DEXs have been observed to trigger more potent major histocompatibility complex class I (MHC I)-restricted cytotoxic T lymphocytes (CTLs) response and maximally activated specific immune responses against HCC (203). In another study, Zhong et al. proved that microwave ablation (MWA) combined with DEXs increased the quantity of CD8+ T cells and decreased the level of FOXP3+ Treg cells in tumor sites compared to MWA alone, thereby reshaping TIME (204). The vivo studies also showed the combination therapy markedly suppressed HCC progression. In a recent study reported by Rezaei et al, DEXs loaded with miR-124-3p mimics reduced the CD4^+^/CD8^+^ cell ratio and FOXP3^+^ Treg/CD8^+^ cells ratio in tumor tissue, eliciting stronger tumor immunosuppression (205). Xiao et al. loaded TEXs into DC cells and inoculated them into PC mouse models in combination with drugs acting on MDSCs (206). The results showed that vaccination with DEXs could markedly prolong mice’s survival time, and co-inoculation triggered more activated T cells and more effective antitumor immune responses. Moreover, exosomes secreted by other cells may also participate in the treatment of GI cancer by regulating immune function. For example, Exosomes from heat-treated malignant ascites of GC patients induced CTL responses via increase of heat shock proteins and maturation of DC cells (207). In brief, exosomes offer a cell-free immunotherapy vaccination option that may be beneficial for treating drug-resistant GI cancer.

## 6 Conclusions and perspectives

Despite the rapid progress in modern medical research, the survival rate of patients with GI tumors has not been significantly improved, and the role of drug resistance cannot be ignored. At the same time, mounting evidence has suggested that exosomes are essential to the occurrence and development of chemotherapy resistance in GI cancer. Exosomes mediate the transfer of several molecules associated with their biogenesis as well as partially affect the communication of GI tumor cells and various cells in the tumor microenvironment. Owing to their inherent stability and accessibility, exosome-derived biomarkers have been rapidly incorporated into clinical practice. Researchers also explored their potential as therapeutic agents, yielding promising outcomes. We provide a comprehensive overview of the pivotal role that exosomes play in the development of drug resistance across diverse GI tumors, while underscoring their utility as diagnostic, prognostic, and therapeutic tools. Nevertheless, there are still several obstacles to overcome before exosomes can be used in the clinical treatment of chemoresistant GI cancer. For example, different isolation methods may result in acquiring varied exosome subpopulations with distinct functions in identical body fluids (208). Currently, numerous techniques exist to isolate and identify exosomes in biological fluids, each with its strengths and weaknesses, but there is still no standardized method. In addition, the time and economic costs of exosome enrichment are so high that the current research is limited to cell and animal experiments, and there are few large-scale multicenter trials (209). Besides, the storage and recovery methods for exosomes require further improvement to ensure their effectiveness (210). Undoubtedly, the mechanism through which exosomes are involved in chemoresistance of GI cancer has only been partially elucidated. More systematic research is necessary to explore the functional content of exosomes in greater detail.

In conclusion, as natural intercellular information carriers, exosomes present a promising avenue for precise prediction and treatment of GI tumors. It is believed that with the further exploration of exosomal heterogeneity and the continuous improvement of isolation and analysis techniques, tumor precision therapy based on exosomes will soon be applied in clinics, benefiting patients with GI cancer.

## Glossary

GI: gastrointestinal
EC: esophageal cancer
GC: gastric cancer
HCC: hepatocellular carcinoma
PC: pancreatic cancer
CRC: colorectal cancer
TME: tumor microenvironment
CAFs: cancer-associated fibroblasts
ESCRT: endosomal sorting complexes required for transport
MVBs: multivesicular bodies
ILVs: intraluminal vesicles
RBPs: RNA-binding proteins
LLPS: liquid-liquid phase separation
PDC D6Ips: programmed cell death 6-interacting proteins
Tsg101: tumor susceptibility gene 101
MHC: major histocompatibility complex
ELISA: Enzyme-Linked Immunosorbent Assay
ExoTIC: Exosome Total Isolation Chip
DLD: deterministic lateral displacement
CTCs: circulating tumor cells
DEP: dielectrophoretic
ACE: alternating current electromotor
ARF: acoustic radiation force
RInSE: Reversible Interaction Nanoscale Sorting Enrichment
SPR: surface plasmon resonance
nPLEX: Nanoplasma exosome
miRNA: micro-RNA
PCR: polymerase chain reaction
MB: molecular beacons
LSPR: localized surface plasmon resonance
HBV: hepatitis B virus
HCV: hepatitis C virus
TP53: tumor protein p53
LRP1B: low-density lipoprotein receptor-related protein 1B
ARID1A: AT-rich interaction domain 1A
KRAS: Kirsten rat sarcoma viral oncogene homologue
APC: adenomatous polyposis coli
TERT: telomerase reverse transcriptase
CTNNB1: β-catenin 1
ESCC: esophageal squamous cell carcinoma
EAC: esophageal adenocarcinoma
M-MDSCs: monocytic myeloid-derived suppressor cells
STAT3: signal transducing activator of transcription 3
NFs: normal fibroblasts
RIG-I: retinoic acid-inducible gene-I
IFN: interferon
PTX: paclitaxel
5-FU: 5-fluorouracil
AMSC: adipose tissue-derived mesenchymal stem cells
DOX: doxorubicin hydrochloride
VAMP2: vesicle-associated membrane protein 2
Treg: regulatory T cells
GEM: gemcitabine
PDAC: pancreatic ductal adenocarcinoma
PSCs: pancreatic stellate cells
GPX4: glutathione peroxidase 4
MMP14: matrix metalloproteinase 14
EMT: epithelial-mesenchymal transition
CSCs: cancer stem cells
GPC3: glypican 3
GEA: gastro-esophageal adenocarcinoma
OS: overall survival
AUC: area under curve
PFS: progression-free survival
DPP4: dipeptidyl peptidase IV
PFS: progression-free survival
TACE: transarterial chemoembolization
RT-qPCR: reverse transcription quantitative polymerase chain reaction
CCDC80: coiled-coil domain-containing protein 80
PAK4: P21-activated kinase 4
miR-21i: miR-21 inhibitor oligonucleotide
ASO: anti-sense oligonucleotide
TDEs: tumor-derived exosomes
REG3β: regenerating islet-derived protein 3 beta
TIME: tumor immune microenvironment
DCs: dendritic cells
DEXs: DC-derived exosomes
MHC I: major histocompatibility complex class I
CTLs: cytotoxic T lymphocytes
MWA: microwave ablation
